# *Necrobia rufipes* (De Geer) Infestation in Pet Food Packaging and Setup of a Monitoring Trap

**DOI:** 10.3390/insects11090623

**Published:** 2020-09-11

**Authors:** Sara Savoldelli, Costanza Jucker, Ezio Peri, Mokhtar Abdulsattar Arif, Salvatore Guarino

**Affiliations:** 1 Department of Food, Environmental and Nutritional Sciences (DeFENS), University of Milan, Via G. Celoria 2, 20133 Milan, Italy; costanza.jucker@unimi.it; 2 Department of Agricultural, Food and Forest Sciences (SAAF), University of Palermo, Viale delle Scienze, Building 5, 90128 Palermo, Italy; ezio.peri@unipa.it (E.P.); mokhtar.arif@unipa.it (M.A.A.); 3 Institute of Biosciences and Bioresources (IBBR), National Research Council of Italy (CNR), Corso Calatafimi 414, 90129 Palermo, Italy

**Keywords:** red-legged ham beetle, packaging, adhesive traps, food attractants

## Abstract

**Simple Summary:**

*Necrobia rufipes* (Coleoptera: Cleridae) is an emerging pest of pet food stores. Information on infestation modalities for this pest is absent and specific monitoring tools are missing. In this paper, the adults’ and larvae’s ability to enter into pet food packaging was evaluated. Furthermore, to set up of a monitoring trap behavioral bioassays were carried out: testing two adhesive surfaces, one generally used in mouse glue trap and the other used in cockroach trap, to evaluate their ability in avoiding insects’ escape; screening different molecules, as candidate food attractants: methyl cyclopentenolone, squalene and stearic acid. The results evidenced that *N. rufipes* enter in packaging through the air vent valves, suggesting that a way to prevent insect infestation would be to modify packaging. Tests showed that the glues have strong differences in the ability to retain the caught insects, with mouse glue more effective than cockroach glue. The behavioral bioassays indicated that methyl cyclopentenolone and squalene are able to attract *N. rufipes* adults in olfactometer. Finally, the dual-choice arena bioassays showed that a mixture of pet food and methyl cyclopentenolone elicited the strongest attraction in *N.* *rufipes* adults, suggesting that this mixture can be used as lure in monitoring traps.

**Abstract:**

*Necrobia rufipes* (De Geer) (Coleoptera: Cleridae), also known as the red-legged ham beetle, is a newly emerging pest of pet food stores, causing apprehension among producers worldwide. Concerns about this pest are exacerbated by the lack of information about infestation modalities in pet food, while specific monitoring tools are missing. Considering that adequate pet food packaging could limit *N. rufipes* infestations, information about the penetration modalities in commonly used pet food packaging is needed. Moreover, the development of appropriate monitoring instruments is urgent to detect pest presence early and to reduce chemical treatments for its control. In this paper, the adults’ and larvae’s ability to enter into pet food packaging was evaluated. Furthermore, to develop monitoring traps, behavioral bioassays were done: (1) testing two different commercial adhesive surfaces, one generally used in mouse glue traps (MG), and the other used in cockroach glue traps (CG), to evaluate their different abilities in avoiding insects’ escape; (2) screening different molecules, typical of the substrates attacked by *N. rufipes*, as candidate food attractants for this pest: methyl cyclopentenolone (MCP), squalene (SQ), and stearic acid (SA). The results show that *N. rufipes* adults and larvae enter into packaging through the air vent valves on the bottom, suggesting that a way to improve the packaging to prevent insect infestation would be to modify these points of weakness. Laboratory tests show that the different bioassayed glues have strong differences in the ability to retain the caught insects, with MG being more effective than CG. The behavioral bioassay indicated that MCP and SQ attract *N. rufipes* adults in olfactometer. Finally, the results of dual-choice arena bioassays show that among the candidate attractant tested, a mixture of pet food (PF) and MCP elicited the strongest attraction in *N. rufipes* adults. These results encourage further experiments with the use of an MG adhesive trap loaded with a mixture of PF+MCP to test the effectiveness of such a tool for monitoring *N.* *rufipes* in pet food industries and warehouses.

## 1. Introduction

Within the pet industry, the pet food market occupies the largest market share, having a value greater than 90 billion US dollars globally in 2018, and it is seen as a valuable market with great growth prospects for years to come [1,2,3]. Pet food products are mainly made from dry cereal products, legumes, oilseeds, and animal by-products in whole or processed form [4], with substrates vulnerable to stored-product pest infestations that in retail stores can cause significant economic loss by contaminating and damaging products on the shelves [5]. The main pests associated with pet food are the Indian meal moth *Plodia interpunctella* (Hübner) (Lepidoptera: Pyralidae) and the coleopterans *Sitophilus* spp. (Dryophthoridae), *Oryzaephilus mercator* (Fauvel) (Silvanidae), *Tribolium castaneum* (Herbst) (Tenebrionidae), *Lasioderma serricorne* (F.) (Anobiidae), *Stegobium paniceum* (L.) (Anobiidae), and *Necrobia rufipes* (De Geer) (Cleridae) [4,6,7,8].

Among these species, the red-legged ham beetle, *N. rufipes*, generally found in stored commodities such as copra (dried coconut), cheese, dried fish, ham, and other products rich in protein content, in recent years has caused increasing damage to pet food products, with growing concerns from producers [8,9,10]. This pest has a worldwide distribution range, and its occurrence has been recorded in many countries of South, Central, and North America; Europe; Asia; and Oceania [9,10,11,12]. Recently, pet food infestations caused by this beetle at retailers have been reported throughout several Mediterranean countries [13]. *Necrobia rufipes* was found in pet food bags in warehouses and stores, contributing to the infestation spreading along the entire chain to the final consumers. In particular, anecdotal reports from the producers indicated that dog food is especially preferred and attacked by this species rather than other types of pet food. In the literature, no data are available about *N. rufipes’* ability to penetrate packaging, neither as penetrators nor as invaders, causing post-production infestation.

Economic losses associated with this clerid species are related to the feeding activities of larvae and adults, as well as cross-infestations, which lead to the reduction in product prices [14]. Larvae of all instars are repelled by light, and at the end of larval stage, they seek a dark and dry spot in which to build their cocoon, which can be completed within 24 h. The cocoon is formed by filling in the open sides of the crevice chosen by the larva for pupation with a white substance vomited from the mouth of the larva in frothy droplets [11]. Adults can fly, and their body length ranges from 3.5 to 7 mm [11]. To date, the common control methods of *N. rufipes* populations mainly rely on chemical treatments [12,15,16]. However, considering that these treatments have increasing restrictions in several countries worldwide and that, in general, insecticides pose safety concerns for workers and a risk of contamination with residues of the final products, new, appropriate strategies for the management of this pest are urgent.

A crucial point that impairs the management of *N. rufipes* is the lack of adequate semiochemical-based tools for specific monitoring, which are essential for its quick detection in pet food retails, warehouses, or industries, as are used for several stored pest species [17,18,19]. To date, the records of *N. rufipes* in multi-capture traps for stored pest insects are rare, probably due to the facultative predator behavior of the red-legged ham beetle toward other captured species [4]. Moreover, incidental observation of adults that fell on sticky traps commonly used for crawling insects showed their ability to escape from the adhesive surface. In this context, the lack of a specific trap for *N. rufipes* monitoring is an obstacle for integrated pest management programs, and a monitoring device to detect its presence and estimate the population increase is strongly needed. Developing an optimal monitoring device is based on the selection of candidate attractants that can bring the insects inside the trap and the screening of the adhesives that can prevent their escape.

The first objective of this study was to verify the red-legged ham beetle’s ability to identify access points of commercial pet food bags, represented by the air vent valves on the bottom, and to enter through them and infest the product inside. The second aim was to set up an appropriate trapping tool for *N. rufipes* monitoring. Behavioral bioassays were carried out by (1) testing different adhesive surfaces to assess their capacity to retain the adults caught in the trap and (2) screening chemical attractant candidates, i.e., volatile components of substrates attacked by this pest, which might be exploited for their use as a lure for *N. rufipes*.

## 2. Materials and Methods

### 2.1. Insect Rearing

A laboratory colony of *N. rufipes* was established and maintained in an environmentally controlled room (25 ± 1 °C, 50 ± 5% relative humidity (RH), photoperiod 16:8, L:D) in plastic cages (25 cm × 25 cm × 40 cm) with two 5 cm diameter mesh-covered holes for ventilation. The colony was fed with a mixture of pet food, dried fish, and ham [20].

### 2.2. Behavioral Tests on Pet Food Packaging

Behavioral experiments to understand how *N. rufipes* larvae and adults can enter into pet food packaging were carried out in order to simulate real-world conditions [21]. Commercial polypropylene-woven (PP) pet food bags (UFlex Ltd., Noida, India) containing 14 kg of pet food with chicken meat for adult dog (Purina-Nestlè, Portogruaro, VE, Italy) were tested. PP bags are heat-sealed, and on the bottom there are 4 air vent valves (2 on each side) with a diameter of 10.5 mm. PP bags were individually placed in a plastic box (78 cm × 56 cm × 43 cm; 130 L) closed with a lid, with *N. rufipes* larvae or adults, which were tested in separate experiments. Thirty II instars larvae or thirty 14-day-old adults (males and females—approx. sex ratio 1:1) were released in the box; 5 replications were carried out both for adults and for larvae. Tests were carried out in a climatic room at 25 ± 1 °C, 50 ± 5% RH, photoperiod 16:8, L:D. The test lasted 2 weeks, after which insects inside packaging were counted. Each PP bag was carefully observed using a stereomicroscope (Leica MZ12, Leica Microsystems GmbH, Wetzlar, Germany) to evidence the presence of any holes made by insects.

### 2.3. Suitability Tests of Adhesives for Traps

The suitability of two commercial adhesive bases to retain *N. rufipes* adults was assessed in laboratory tests. A glue generally used in mouse traps (MG) (Ecotrap Quadro inPEST P-02013, GEA S.r.l., Settimo Milanese, Milan, Italy) and the other used in cockroach traps (CG) (Geotrap Gel inPEST P-04047, GEA S.r.l., Settimo Milanese, Milan, Italy) were distributed on a cardboard base of 10.5 cm × 9.5 cm, designed to be used in a monitoring device of 12.8 cm wide, 12.6 cm long, and 5.0 cm in height (Rufy trap water proof; GEA S.r.l., Settimo Milanese, Milan, Italy) (Figure S1). At the center of the adhesive base, a silicone paper disc (1.5 cm Ø) was placed and used as adult release point. A single *N. rufipes* adult was put on silicone paper disc, and the detachment or the distance traveled by adults on the adhesive surface was registered at regular intervals of 1, 2, 3, 6, and 24 h and subsequently once a day until all adults escaped or died. The ability of the adhesive bases to prevent the *N. rufipes* adults’ escape was estimated by measuring the distances traveled on the glue at different times. Measurements of distance from the release point were made using a digital caliper. Moreover, the number of total escapes was counted at different times of observation. Thirty replicates were carried out for each adhesive at room condition of 22 ± 1 °C and 50 ± 5% RH.

### 2.4. Bioassays with Food Attractants 

#### 2.4.1. Four-Choice Pitfall Olfactometer Bioassays 

The response of *N. rufipes* adults to the chemicals tested as attractant candidates were evaluated using a four-choice olfactometer. The candidate chemical attractants were selected for their occurrence in the substrates commonly infested by *N. rufipes* as foodstuff, dried fishes, and carcasses [22,23,24]. Tested chemicals were as follows: 3-Methylcyclopentane-1,2-dione (>99% pure, BBE Int., Lodi, Italy), henceforth called methyl cyclopentenolone (MCP); stearic acid (SA); and squalene (SQ) (both >99% pure, Sigma-Aldrich, Milan, Italy). The olfactometer consisted of a steel arena (20 cm Ø × 5 cm height) with four diametrically opposed holes (2 cm Ø) on the bottom located 3 cm from the sidewall. A 40 mL glass flask with paraffin oil-coated neck was positioned under each hole. The tested attractants were diluted in acetone at 25,000 ppm. An aliquot of 400 µL was pipetted on filter paper to achieve the dose of 10 mg, and after 5 min, to allow solvent evaporation, it was placed inside one of the glass flacks. The control flask was loaded with the same volume of acetone. The internal border of each flask was gently brushed with paraffin oil to prevent the insects from returning to the arena. For each replication, five insects (males or females) were placed in the center of the arena under a clean glass cup (180 mL) and allowed to acclimate for 30 min. Then, the glass cup was removed, and the arena was covered with a steel lid to allow ventilation and prevent insect escape. The duration of each replication was 24 h at a room condition of 22 ± 1 °C and 65% RH. After each trial, the arena was carefully washed with soap and rinsed throughout with tap water and acetone. Eighteen replications were performed for each assay, for a total of 90 insects tested; each individual did not undergo more than one trial. Non-responder insects, i.e., the insects that remained in the steel arena without making a choice, were not considered for the statistics. The position of the chemical stimuli and the control was randomized for each replication.

#### 2.4.2. Dual-Choice Arena Bioassays

The dual-choice arena bioassays were carried out in Plexiglas cages 50 cm × 30 cm × 30 cm high with the two lateral opposite sides of the cage made of net for ventilation. Candidate attractants were placed inside two sticky cardboards (50 cm apart) of the “Rufy trap water proof” model. Each trap’s sticky surface was loaded with the glue that best performed the insect escape avoidance in the latter experiment. Adult bugs were not starved before the experiments. Individuals (n = 5 per replication) were released into the cage at 9:00 AM, and the total number of insects on each sticky trap was recorded after three days. Sixteen to twenty-three replications were carried out per experiment. The non-responder insects, i.e., the insects that remained in the arena without entering in one of the traps, were removed and not considered for the statistics. The position of the treatments inside the arena was alternated after each replicate. The tested attractant was placed at the center of the adhesive surface of the trap in a plastic container (3 cm Ø). After each bioassay, the Plexiglas cage was cleaned with water and dried. Observations were conducted in static air under controlled laboratory temperature and humidity conditions (25 ± 2 °C, and 50 ± 15% RH). A first bioassay was carried out to validate the instrument, testing a trap loaded with 2 g of pet food for adult dogs of medium-size Purina pro-plan (Purina-Nestlè, Portogruaro, VE, Italy) versus an unloaded trap. The pet food ingredients were chicken (20%), wheat, dehydrated poultry protein, maize, rice (7%), animal fat, dried beet pulp, soya meal, digest, maize gluten meal, minerals, dried egg, fish oil; the chemical composition, according to the producer, was protein 26%, fats 16%, crude ash 7.5%, fibers 2.5%. The following candidate attractant chemicals were successively tested: MCP, SA, and SQ as in the four-choice olfactometer experiments. Candidate attractants were tested alone at the doses of 0.2 g versus 2 g of pet food as positive control. Furthermore, the tested chemicals (0.2 g) were tested mixed with 2 g of pet food versus the same amount of pet food alone.

### 2.5. Statistical Analysis

The data of the tests with food packaging, i.e., individuals (larvae or adults) found inside the packaging, were analyzed by t-test for independent samples. The data obtained from the test of adhesives for traps, i.e., the distances traveled in the glued cardboard from the *N. rufipes* adults at different times of observations, were analyzed by t-test for independent samples. Data of four-choice olfactometer experiments, i.e., the mean number of adults inside the treated flask after 24 h, were compared using a one-way ANOVA, followed by Tukey’s test. The data obtained from the dual-choice arena bioassays were analyzed using t-tests. All statistical analyses were performed using Statistica 10.0 for Windows.

## 3. Results

### 3.1. Behavioral Tests on Pet Food Packaging

Tests on packaging showed that *N. rufipes* larvae and adults enter commercial PP bags, since individuals were recorded in each tested bag. No differences between larvae and adults in the aptitude to enter the packaging were observed, with a mean number (±SE) of 9.2 ± 2.2 adults and 11.4 ± 2.8 larvae (*t* = 0.63; df = 8; *p* = 0.54). Careful stereomicroscope observations of the PP packaging did not evidence the presence of holes of penetration made by insects. Consequently, we can assume that the only entry point exploited by the *N. rufipes* adults and larvae was the presence of the air vent valves on the bottom of the packaging (Figure S2). 

### 3.2. Suitability Test of Adhesives for Traps

**Figure 1:**
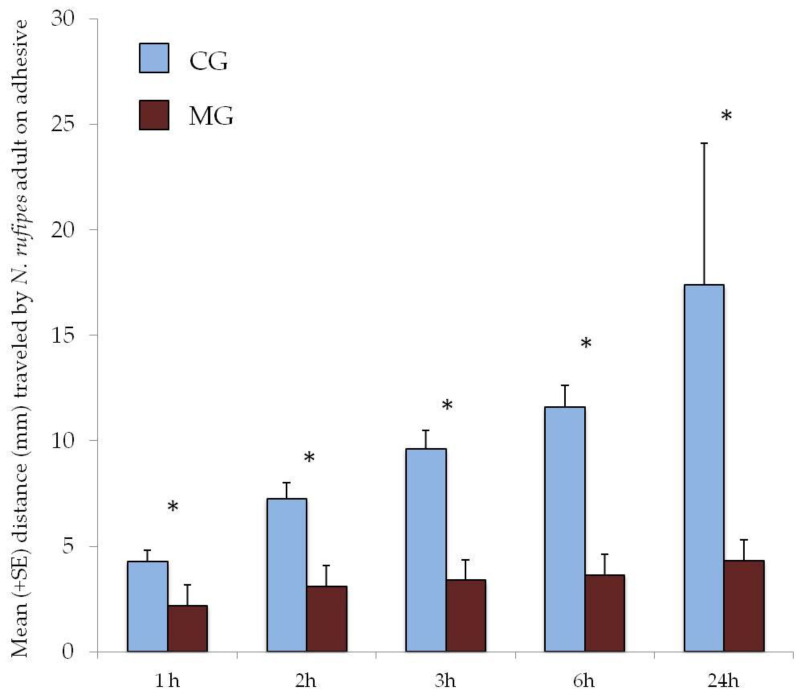
Mean distance (mm + SE) traveled by *Necrobia rufipes* adult on cockroach glue (CG) and mouse glue (MG) bases. The means were calculated considering only the retained adults (not considering the escaped ones); therefore, for CG base, n = 30 (1 h), 29 (2 h), 27 (3 h), 25 (6 h), 5 (24 h); and for MG base, n = 30 (1 h, 2 h, 3 h, 6 h, 24 h). * = *p* ≤ 0.001.

**Figure 2:**
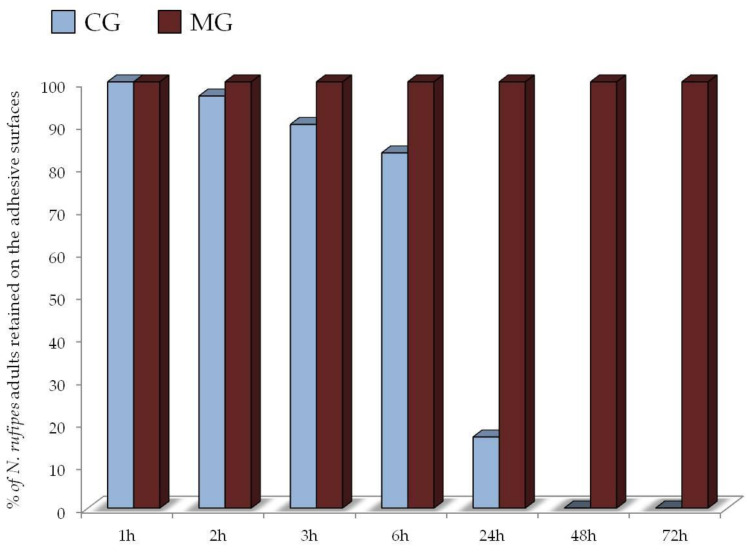
Percentage of *Necrobia rufipes* adults retained on cockroach glue (CG) and mouse glue (MG) bases after 1 h, 2 h, 3 h, 6 h, 24 h, 48 h, 72 h.

Tests on adhesive surfaces lasted up to 72 h, when we verified that all adults had escaped or died. The mean distance traveled by *N. rufipes* adults on the two adhesive surfaces at different times of observation is reported in Figure 1. Tested glues showed strong distinction in the ability to retain the caught insects, with MG performing better than CG already after 1 h of testing (*t* = 3.30; df = 58; *p* = 0.001) (Figure 1). Significant differences were observed again in the observations carried out at 2 h (*t* = 4.86; df = 57; *p* < 0.001), 3 h (*t* = 6.48; df = 55; *p* < 0.001), 6 h (*t* = 7.08; df = 53; *p* < 0.001), and 24 h (*t* = 4.31; df = 57; *p* < 0.001). On CG, the mean distance traveled by *N. rufipes* adults increased over time (from 4.27 ± 0.53 mm after 1 h to 17.36 ± 6.73 mm after 24 h), while on MG, it remained almost unchanged (from 2.18 ± 0.34 mm after 1 h to 4.31 ± 0.67 mm after 24 h). A significant majority of *N. rufipes* adults were able to escape the CG base already after 24 h, with a mean percentage of individuals retained on glue of 16.7% (Figure 2). All insects were able to escape from the CG base within 48 h, while no adults broke free from MG adhesive until the end of the test, after 72 h (Figure 2).

A careful observation of adults during the test showed how they were able to use their jaws to free their glued legs from the adhesive surface of the CG base (Video S3). In this way, they managed to advance rapidly until they were freed, either by crossing the entire adhesive area or by flying away.

In contrast, in the MG base, *N. rufipes* adults were unable to break free until the end of the trial (72 h). We observed that, when the insect tried to detach itself, the glue strands kept it attached (Video S4). Even their jaws and antennae often remain glued when the insect tried to free itself, and they were able to travel at most 4.31 ± 0.67 mm after 24 h (Figure 1). At the end of the trial (72 h), *N. rufipes* adults traveled a distance of 5.04 ± 0.75 mm, with no adult escapes.

### 3.3. Bioassays with Food Attractants 

#### 3.3.1. Four-Choice Pitfall Olfactometer Bioassays

**Figure 3:**
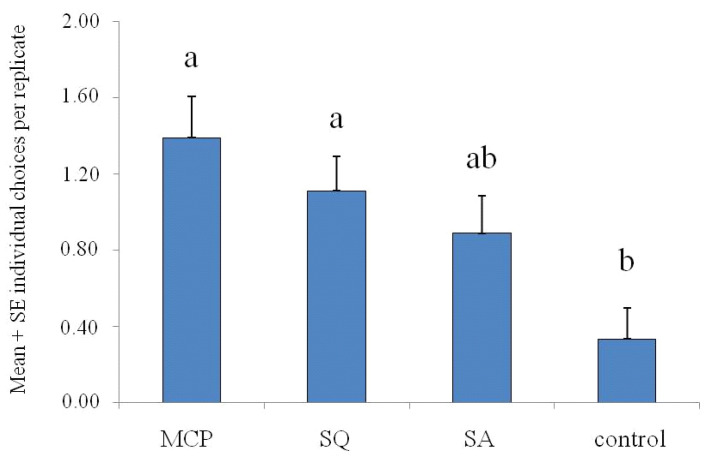
Four-choice olfactometer bioassays: mean (+SE) responses of *Necrobia rufipes* adults to the chemicals tested; control = empty flask; MCP = methyl cyclopentenolone; SQ = squalene; SA = stearic acid. Different letters indicate statistically significant differences (*p* < 0.05) among the treatments (one-way ANOVA, followed by Tukey’s test).

The response of *N. rufipes* adults to the chemicals tested as candidate attractants in the four-choice olfactometer is reported in Figure 3. Overall, 72 insects responded out of the 90 insects tested in total. Adult beetles’ response toward the different odor sources in four-choice olfactometer was significantly different among treatments (df = 3; *F* = 5.60; *p* < 0.01 *).

Among the tested chemicals, the MCP and SQ attracted a higher number of individuals inside the treated flask in comparison to the control (*p* = 0.02) (Figure 3). In contrast, SA was not able to attract a number of individuals statistically significantly different from the control (*p* = 0.17) (Figure 3). Finally, no statistically significant differences were recorded among the three chemicals tested (*p* = NS).

#### 3.3.2. Dual-Choice Arena Bioassays

**Figure 4:**
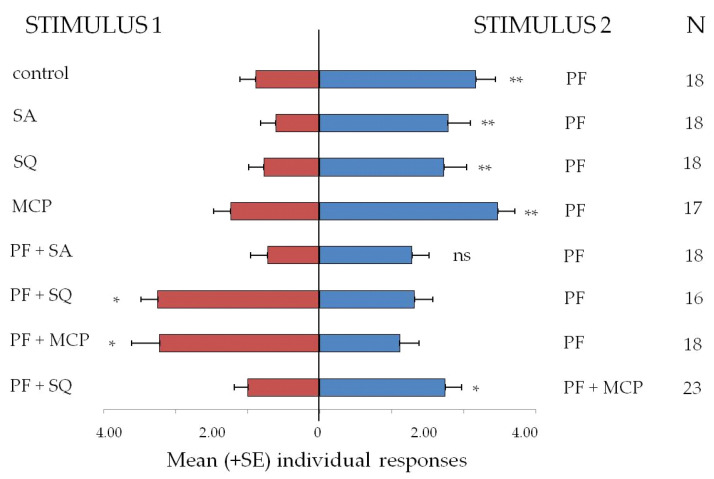
*Necrobia rufipes* adult responses (mean + SE) in dual-choice arena toward the stimuli tested inside the traps and their combinations. Control = empty; PF= pet food; MCP = methyl cyclopentenolone; SQ = squalene; SA = stearic acid; N = number of responders; * = *p* < 0.05; ** = *p* < 0.01.

Responses of *N. rufipes* adults in dual-choice arena bioassays are shown in Figure 4. Overall, out of the 730 insects tested in this bioassay, 431 (59.04% of the total) made a choice entering inside the trap, while 299 remained in the arena. In the first experiment, carried out to validate the instrument, adults were more attracted inside the trap loaded with the pet food (PF) than in the empty trap used as control (*t* = 3.97, df = 17, *p* < 0.01). The bioassays testing the candidate attractants alone versus the pet food indicated a preference response for the pet food rather than for SA (*t* = 3.37, df = 17, *p* < 0.01), SQ (*t* = 3.07, df = 17, *p* < 0.01), and MCP (*t* = 3.34, df = 16, *p* < 0.01). However, when candidate attractants were tested mixed with the pet food, two of them elicited a stronger attraction than pet food alone inside the traps: SQ (*t* = 2.45, df = 17, *p* < 0.05) and MCP (*t* = 2.20, df = 17, *p* < 0.05). In contrast, the mix of SA with pet food did not attract more individuals inside the traps than pet food alone (*t* = 1.70, df = 17, *p* = ns). Finally, the mix of pet food with MCP attracted more individuals inside the traps than the mix of pet food with SQ (*t* = 2.55, df = 22, *p* < 0.05).

## 4. Discussion

In recent years, the clerid beetle *N. rufipes* has been increasingly reported as a pest associated with pet food [4,8,9,10]. The investigations carried out in this study give, for the first time, important information on the behavior of this species related to post-production infestation of pet food packaging and provide the basis for a monitoring device for this pest. Our laboratory tests on PP commercial packaging evidenced the ability of *N. rufipes* adults and larvae to enter them, facilitated by the presence of air vent valves, whose size allowed adult and larva to enter. Tests performed resembling real-world conditions demonstrated that *N. rufipes* can identify access points of bags (hidden at the bottom), probably driven by pet food odor. In the literature, there are no data on the ability of this pest to enter packaging, and the results of this study represent the first preliminary information about this. We did not observe on tested bags any damages or signs of boring activity made by insects, suggesting entrance in packaging is only determined by the exploitation of the air valves by *N. rufipes* larvae and adults. In any case, we cannot evaluate the ability of *N. rufipes* to perforate packaging as the presence of air valves might make unnecessary any boring activity, and future tests must be performed to verify it. Generally, stored-product pests are classified as penetrators and invaders, since they come into the packaging through small holes already present or can actively penetrate various packaging materials [21,25]. Several packaged products have openings to accommodate pressure differences and facilitate stacking, as in the case of the PP bags tested in this study. However, as they release odors, openings can represent a source of attraction for stored-product pests that can also use them as a point of entry into the foodstuff. Some insects, such as the Indian meal moth *P. interpunctella*, lay their eggs near the openings so the newborn larvae can enter the packaging; others can easily enter due to their small size, such as *Oryzaephilus* sp.; still others can enlarge the holes to penetrate, such as *Sitophilus* sp. [26,27,28,29]. Data from our tests showed that in the presence of existing holes, *N. rufipes* larvae and adults behaved like invaders. Future tests will focus on verifying whether or not *N. rufipes* larvae and adults can bore through packaging in the absence of openings. What has been observed so far suggests that by improving the weakness point of packaging, i.e., by modifying the current air vent valves, *N. rufipes* infestations could be reduced. 

The results obtained from the behavioral experiment to set up a first monitoring tool device for *N. rufipes* give an important indication about both the adhesive material and the attractant semiochemicals that can be utilized for this pest. The experiment carried out with different adhesive surfaces indicated that MG is more effective than CG for retaining the captured insect in the traps, preventing their escape. Although CG surfaces are considered easier and cleaner to use than MG [30,31], our results discourage their use in *N. rufipes* traps. There are few examples in the entomological literature of experiments elucidating the different performances of glues for insect sticky traps. Yee [30] compared the ability to retain the captured *Rhagoletis* spp. (Diptera: Tephritidae) fruit flies in the traps by two different kinds of glue, mentioned as sticky gel (SG) and hot-melt pressure-sensitive adhesive (HMPA). Moreover, Lo et al. [31] evidenced that an HMPA performed better for larger insects (>1.5 mm), while an SG adhesive tended to be more effective for smaller insects. In our observations, we evinced that the ability of *N. rufipes* to break free from CG adhesive surface seems to be determined not only by its physical characteristics but also by their ability to use their jaws to free their glued legs from the adhesive surface of the hot melt cardboard. 

The data obtained from behavioral bioassays with candidate food attractants indicate that MCP and SQ elicit attraction behavior in *N. rufipes* adults. In the four-choice olfactometer bioassays, these two compounds elicited attraction on their own in comparison with the solvent. MCP, also known as cyclotene or maple lactone, is a pronounced odorant of natural maple syrup. This compound has already been reported as a food attractant for other foodstuff pests such as cockroaches [23,32]. MCP is a volatile molecule present in several foods and is a component of their flavor, producing a typical caramel smell like odor; in fact, it is present in the profile of the volatile compounds from heated glucose [33]. Furthermore, MCP has also been reported among the volatiles of beef [34], one of the most attractive sources for *N. rufipes* adults, an insect also found in carcasses. Similarly, SQ has also been found in carcasses [35], and, similarly to MCP, elicited attraction toward *N. rufipes* adults in our study. Moreover, SQ often occurs in dried fish [36], another substrate attacked by this pest [37].

However, our experiment in the dual-choice open arena demonstrated that MCP and SQ were less attractive in comparison to pet food. This is not surprising, considering that the attraction toward food and oviposition sites in insects is often mediated by a complex of volatiles, such as the ones emitted by pet food, rather than by one key odorant molecule. 

On the other hand, both MCP and SQ added to the pet food enhanced the *N. rufipes* adult attraction to this source in comparison to pet food alone, indicating the responsiveness of *N. rufipes* to these chemicals. In particular, the mixture MCP and pet food caused a higher attraction in comparison with SQ plus pet food, suggesting that it can be considered the best candidate among the tested attractants for being used for further field testing experiments as a lure for *N. rufipes* monitoring traps. It is also to be stated that previous research on the aroma of dog food did not evidence the presence of MCP or SQ among the volatiles of such substrates [38,39]. Further trials will focus on evidence of whether traps loaded with the mixture of such chemicals with pet food can actively attract *N. rufipes* individuals in environments rich with background odor stimuli, such as pet food warehouses and retail stores. 

To our knowledge, this is the first report of a specific lure for *N. rufipes* monitoring, a tool highly necessary to promptly identify its presence in facilities, warehouses, and retail stores, particularly considering that the concealed behavior of this pest in such environments makes it difficult to detect its presence early, before that infestation of pet food occurs. In fact, mature larvae and pupae, protected within their white cocoons and hidden in cracks and crevices, are not easy to observe. The cocoons have been found on wooden pallets on which pet food infested by *N. rufipes* was transported (Savoldelli, personal observation). It is likely that these pallets, reused for the transport of new goods, become the way of spreading the infestation. This aspect is not to be overlooked and, to avoid the spreading of infestation, a careful inspection of pallets coupled with the use of monitoring devices such as the MG traps loaded with PF+MCP should be considered to detect the infestation early. Future work will focus on field tests of such a monitoring device loaded with PF+MCP to estimate the effectiveness of such tools in contexts as pet food storage and warehouses.

## 5. Conclusions

The results obtained in this study indicate that the design of an adequate packaging can help to prevent or reduce *N. rufipes* infestation of pet food. Similarly, an adequate trap projecting is crucial to develop a tool for appropriately monitoring this pest. In this context, the choice of an adhesive that can successfully prevent trap escape is the first step, and our results demonstrate that MG can completely hold adults back, while CG is unsuitable as the insects can break free from the trap. Finally, the bioassays with candidate attractants evidenced an attraction response of the adults to MCP and SQ and that a mixture of PF+MCP can be considered a first specific lure for *N. rufipes*, suitable for further field tests.

## Supplementary Materials

The following are available online at https://www.mdpi.com/2075-4450/11/9/623/s1, Figure S1: “Rufy trap water proof” by GEA S.r.L., Settimo Milanese Milan, Italy; Figure S2: Air vent valves on the bottom of the PP packaging, Video S3: *N. rufipes* adult on CG base, Video S4: *N. rufipes* adult on MG base.
