# Supplementary figures and images for: Necrobia rufipes (De Geer) Infestation in Pet Food Packaging and Setup of a Monitoring Trap
Source: Insects. 2020 Sep 11;11(9):623. doi: 10.3390/insects11090623 (PMC7565875; doi:10.3390/insects11090623)

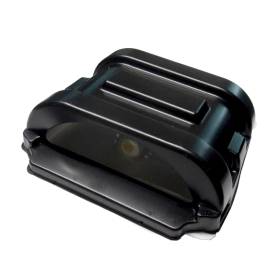

Supplement: Supplementary file 1 [file insects-11-00623-s001.zip › Figure_S1.jpg]

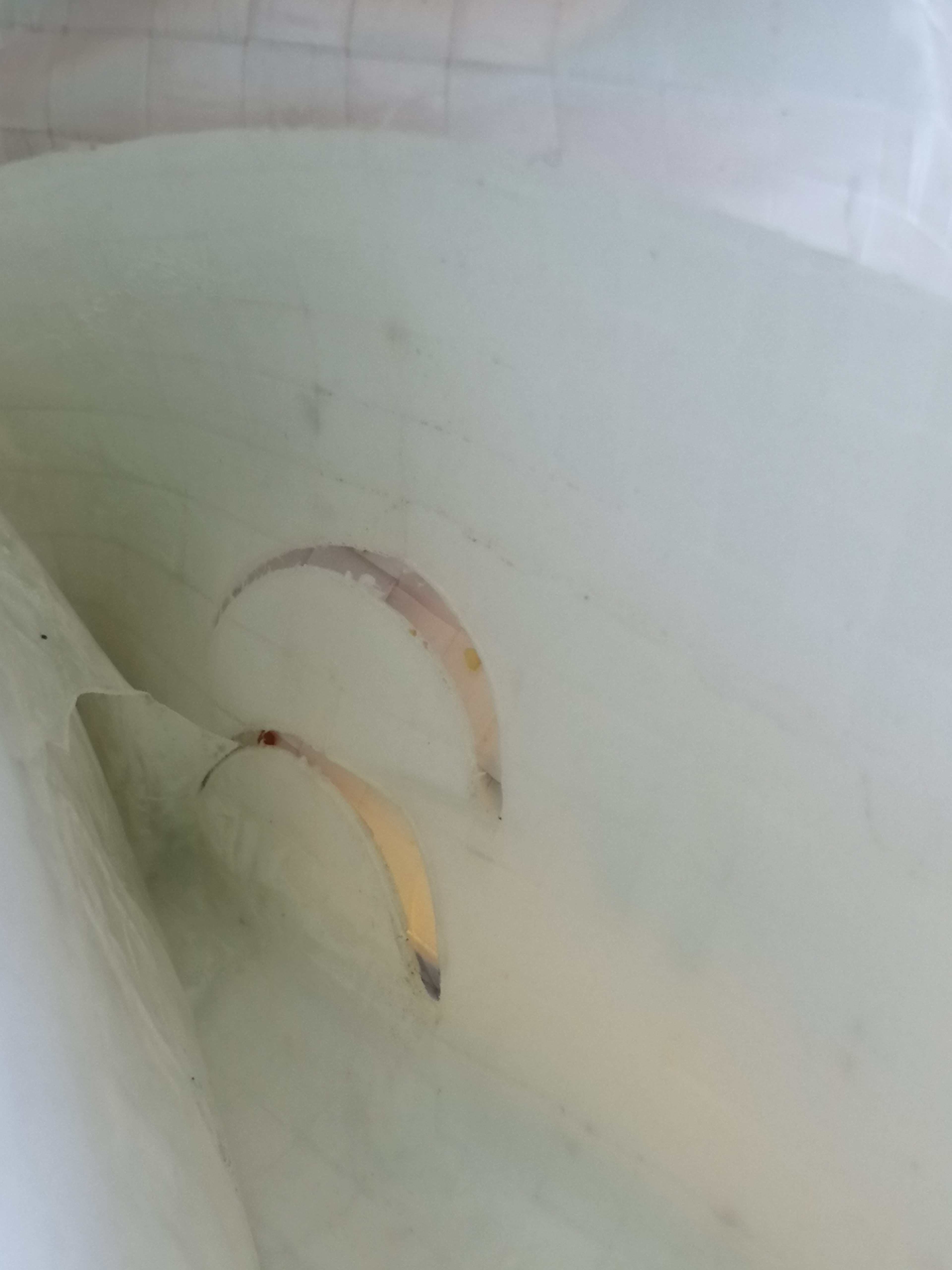

Supplement: Supplementary file 1 [file insects-11-00623-s001.zip › Figure_S2.jpg]
